# Analysis of the duodenal microbiotas of weaned piglet fed with epidermal growth factor-expressed *Saccharomyces cerevisiae*

**DOI:** 10.1186/s12866-016-0783-7

**Published:** 2016-07-28

**Authors:** Zhongwei Zhang, Lili Cao, Yan Zhou, Shujin Wang, Lin Zhou

**Affiliations:** 1 Department of Intensive Care Unit, West China Hospital, Sichuan University, Chengdu, Sichuan 610041 People’s Republic of China; 2 Human and Animal Physiology, Wageningen University, Wageningen, 6700 AH The Netherlands; 3 Shenzhen Premix Inve Nutrition Co., LTD, Shenzhen, 518103 People’s Republic of China; 4 Medical School, Chengdu University, Chengdu, Sichuan 610041 People’s Republic of China

**Keywords:** Bacterial community, Full-length 16S rRNA, *Saccharomyces cerevisiae*, Epidermal growth factor, Weaned piglet

## Abstract

**Background:**

The bacterial community of the small intestine is a key factor that has strong influence on the health of gastrointestinal tract (GIT) in mammals during and shortly after weaning. The aim of this study was to analyze the effects of the diets of supplemented with epidermal growth factor (EGF)-expressed *Saccharomyces cerevisiae* (*S. cerevisiae*) on the duodenal microbiotas of weaned piglets.

**Results:**

Revealed in this study, at day 7, 14 and 21, respectively, the compositional sequencing analysis of the 16S rRNA in the duodenum had no marked difference in microbial diversity from the phylum to species levels between the INVSc1(EV) and other recombinant strains encompassing INVSc1-EE(+), INVSc1-TE(−), and INVSc1-IE(+). Furthermore, the populations of potentially enterobacteria (e.g., *Clostridium* and *Prevotella*) and probiotic (e.g., *Lactobacilli* and *Lactococcus*) also remained unchanged among recombinant *S. cerevisiae* groups (*P >* 0.05). However, the compositional sequencing analysis of the 16S rRNA in the duodenum revealed significant difference in microbial diversity from phylum to species levels between the control group and recombinant *S. cerevisiae* groups. In terms of the control group (the lack of *S. cerevisiae*), these data confirmed that dietary exogenous *S. cerevisiae* had the feasibility to be used as a supplement for enhancing potentially probiotic (e.g., *Lactobacilli* and *Lactococcus*) (*P* < 0.01), and reducing potentially pathogenic bacteria (e.g., *Clostridium* and *Prevotella*) (*P* < 0.01).

**Conclusion:**

Herein, altered the microbiome effect was really *S. cerevisiae*, and then different forms of recombinant EGF, including T-EGF, EE-EGF and IE-EGF, did not appear to make a significant difference to the microbiome of weaned piglets.

**Electronic supplementary material:**

The online version of this article (doi:10.1186/s12866-016-0783-7) contains supplementary material, which is available to authorized users.

## Background

During and shortly after weaning, the immature gastrointestinal tract (GIT) of the mammal has to adapt to the solid diets and bacterial colonization, and then leading to the mucosal surface stimulation from both bacterial antigens and dietary. Thus, the mammal at weaning will be susceptible subjected to infection, disorders and diarrhea, and resulting post-weaning maldigestion and malabsorption [1]. Indeed, weaning stress involves multiple factors, such as the environmental, dietary and physiological stress, which interferes with the intestinal development and post-weaning growth [2]. The changes in the diets, use of the antibiotics, and the intestinal colonization have also likely damaged the intestinal microbial communities, and increased the prevalence of the disorders and diarrhea after weaning [3]. Thus, the homeostasis exists among members of the microbiota, such that the potential pathogenic and non-pathogenic bacteria, have strong influence on the regulation of the intestinal microbiota during and shortly after weaning [4, 5]. Growing evidence has shown that the microbiota of GIT in mammal is a complex biological system, which comprises a vast repertoire of the microbes with considerable the metabolic activity in regard to both the bacterial growth and host health [6]. In the meanwhile, the GIT of the mammals is a reservoir to 10^13^ to 10^14^ commensal bacteria composed of thousands of species that can benefit the host by supplying the nutrients, metabolizing otherwise indigestible food, and preventing colonization by the pathogens [7]. Therefore, to minimize contact between the luminal microorganisms and the intestinal epithelial cell surface is an ideal strategy for maintaining homeostasis with the microbiota of the GIT [8, 9]. In recent years, there has been an explosion of interest to focus on the modulation of intestinal microbiota and host inflammatory responses by the probiotic. In fact, probiotics are defined as the live micro-organisms when administered in sufficient amounts offer the health benefit to its host (Food and Agriculture Organization of the United Nations & World Health Organization, 2002). *Lactobacillus GG*, *Lactobacillus acidophilus*, *Bifidobacterium bifidum*, and *Enterococcus faecium* are typically found in probiotic products for human or animal, as well as yeast, such as *Saccharomyces cerevisiae* (*S.cerevisiae*) [4, 5, 10–12]. Actually, such microorganisms have been used extensively for both prevention and treatment of various inflammatory and infectious intestinal disorders, such as pouchitis [13], inflammatory bowel disease [14], and infectious diarrhea in children [15], and animal models (e.g., rats and piglets) [11, 16]. These findings also further support that these probiotics may provide an approach for the management of weaning stress induced the intestinal dysfunction.

In addition, the dramatically decreased intake of epidermal growth factor (EGF) may also be an important cause of reducing mucosal defenses and increasing weaning stress [17]. Indeed, EGF has many trophic effects on the proliferation and differentiation of epithelial cells in the GLT [18, 19]. Accumulating evidences have also indicated that an exogenous EGF supplement may be effectively taken up by weaned rat or weaned piglet [10, 11, 18, 19]. Actually, the combination of EGF delivery and a probiotic approach has been applied to formulate the dietary supplements for the rats and piglets after weaning [11, 18].

In recent years, the approaches to decrease weaning stress through feeding management and particular nutrients supplement are becoming an interesting issue [10, 11]. Unfortunately, there is no evidence to consider the effect of dietary recombinant *S.cerevisiae* on microbial communities of the GIT, which is characterized using full-length 16S rRNA compositional sequencing. Actually, the majority of the microbes in the GIT are unculturable with routine culture methods. In the last decades, different techniques, such as ITS typing, ARISA, TGGE, DGGE SSCP, T-RFLP and long-PCR-RFLP, have been applied to overcome the problem via analyzing the16s rRNA gene sequence [20, 21]. Although these different methods have facilitated the identification of the microbes residing in some complex ecosystems, another extensively accepted methods in microbial taxonomy research is the 16 s rRNA gene in accordance with the classification in the recent research. Furthermore, along with the appearance of the metagenomic techniques, together with the 16 s rRNA amplicon pyrosequencing, it is possible to detect the composition of both uncultured and cultured and uncultured phylotypes present in any complicated ecosystem [20].

In the current research, the effects of weaned piglet diets supplemented with tagged EGF (T-EGF), extracellular EGF (EE-EGF), and intracellular EGF (IE-EGF)-expressed *S. cerevisiae* on duodenal bacterial community of full-length 16S rRNA using high-throughput sequencing on PacBio® RS II platform, respectively.

## Methods

### Production of recombinant *S. cerevisiae*-expressing tagged EGF, extracellular EGF and intracellular EGF

T-EGF, EE-EGF, and IE-EGF protein-expressed *S. cerevisiae* was designated as the strain of INVSc1-TE(−), INVSc1-EE(+), and INVSc1-IE(+), respectively. In the meanwhile, the empty vector backbone-expressed *S. cerevisiae* was designated as the strain of INVSc1(EV) (as the *S. cerevisiae* control). The strains of INVSc1-TE(−), INVSc1-EE(+), INVSc1-IE(+), and INVSc1(EV) were generated and cultured in accordance with previously described [11].

### Animal experiments

The animal procedures that were used in the current research were based on the guidelines of the China Animal Protection Association, and all of the work was approved by the Shenzhen Animal Care Committee.

#### The piglets and treatment groups

In total of 200 piglets (obtained from the University of Guelph Arkell Swine Research Station,) weaned at day 26, obtained from the Shenzhen Premix Inve Nutrition Co., LTD, were randomly assigned to following 5 groups that were provided: 1) the basal diet per-kg supplemented with 50 mL of fresh media (Control), 2) empty vector-expressed *S. cerevisiae* [INVSc1(EV)], 3) tagged EGF (T-EGF)-expressed *S. cerevisiae* [INVSc1-TE(−)], 4) extracellular EGF (EE-EGF)-expressed *S. cerevisiae* [INVSc1-EE(+)], and 5) intracellular EGF (IE-EGF)-expressed *S. cerevisiae* [INVSc1-IE(+)]. Thus, forty piglets were assigned to each group, and four pens (as an experimental unit) per group and ten piglets per pen. The piglets of each pen had an average initial body weight (BW) of between 6.37 and 6.39 kg.

The concentration of T-EGF, EE-EGF, and IE-EGF protein-expressed *S. cerevisiae* was approximately 30.00 mg/L in accordance with previously described, respectively [11]. Throughout the 21-day trial, the diets per-kg of the INVSc1(EV), INVSc1-TE(−), INVSc1-EE(+), and INVSc1-IE(+) groups were supplemented with 50 mL of the fresh culture from the INVSc1(EV), INVSc1-EE(+), INVSc1-TE(−), and INVSc1-IE(+) strains, respectively. In the meanwhile, the control group was only given fresh media (the same volume). Thus, each piglet in treatment groups was delivered as approximately 2 × 10^8^ live microbes (*S. cerevisiae*) and 600.00 *μ*g EGF per day.

In addition, the diet (Additional file 1: Table S1) was formulated as a powder form without any in-feed antibiotics in accordance with the guidelines of National Research Council (NRC, 2012) [22] for 5- to 10-kg piglets, and contained the similar nutrient levels but differed in terms of whether they contained T-EGF, EE-EGF, or IE-EGF-expressed *S. cerevisiae*, respectively.

The piglets had *ad libitum* access to the water and feed, and then remaining feed was weighed at 0800 h per day. Average daily feed intake (ADFI) and body weight (BW) were recorded weekly to assess the feed-to-gain ratio (F/G) and average daily gain (ADG) and. In addition, the signs of diarrhea, sickness, and abnormal behavior were also recorded daily.

#### The samples collection and processing

At day 0, 7, 14, and 21, respectively, two piglets (approach to average BW in each pen) from each pen were chosen to be killed with the sodium pentobarbital (approximately 50.00 mg/kg.BW) using intravenous injection to sample tissues of the duodenum at an approximately 10-cm section beginning from the pylorus. Furthermore, the entire duodenal contents were rapidly removed with the sterile 1× phosphate buffer saline (PBS) to store at liquid nitrogen for further analysis.

#### Sequence processing and analysis

The full-length 16S rRNA genes of all samples in this study were assessed using high-throughput sequencing on PacBio® RS II platform (Majorbio Bio-Pharm Technology Co., Ltd., Shanghai, China). Briefly, the total DNA of the duodenal content was extracted using the E.Z.N.A.® Stool DNA Kit (E.Z.N.A.® Stool DNA Kit) according to the manufacturer’s protocol. To assess the taxonomic compositions of bacterial community in duodenum, the full-length 16S rRNA gene primers (27 F: 5’-AGAGTTTGATCCTGGCTCAG-3’ and 1492R: 5’-GGTTACCTTGTTACGACTT-3’) were designed for the amplification and subsequent pyrosequencing of the PCR products [23]. The PCR was carried out in triplicate 50 mL reactions with 10 mL 5-fold reaction buffer containing 0.40 mM each primer, 25 ng DNA template, 0.25 mM dNTPs, and 2.50 U Pfu polymerase (TransGen Biotech Co., Beijing, China). The applied PCR conditions were as follows: 94 °C (4 min), followed by 25 cycles of 94 °C (30 s), 55 °C (30 s), and 72 °C (30 s), with a final extension at 72 °C (10 min). The products of PCR were detected using the agarose gels (2 % in TBE buffer), and then purified with DNA gel extraction kit (Axygen Co., China) for later sequencing.

Prior to sequencing, DNA concentration of PCR product was assessed with Quant-iT PicoGreen double-stranded DNA assay (Invitrogen Co., USA), simultaneously was quality controlled using Agilent 2100 bioanalyzer (Agilent Co., USA). The detection of full-length 16S rRNA gene was performed on PacBio® RS II platform (Majorbio Bio-Pharm Technology Co., Ltd., Shanghai, China). The end fragments were blunted and tagged on both a short four-nucleotide sequence (ATCG) and ends with ligation adapters containing a unique 10-bp sequence. As for the sequencing key, four-nucleotide sequence (ATCG) was recognized by the priming sequences and system software. The primer sequences were eliminated after alignment, and then sequences under 200-bp length or of low-quality were also excluded from pyrosequencing-derived data sets. The matrices of DNA distance matrices were analyzed to assess operational taxonomic units (OTUs) at 3.0 % sequence divergences in our communities. In the meanwhile, the abundance of coverage estimator (ACE), the bias-corrected Chao1 richness estimator, the coverage percentage, and the diversity indices of Shannon and Simpson were also calculated in this study. All analyses were performed by MOTHUR program (see http://www.mothur.org).

### Statistical analysis

The relative abundances of genera and species level higher than 0.5 % within total bacteria were defined as the predominant genera and species, and sorted for further analysis, respectively. To assess microbial composition after supplement with recombinant *S. cerevisiae*, the ratio of relative abundance of one bacterial group in treatment groups to its relative abundance in the control group was analyzed on genus and species level, respectively. In the current research, the abundance of OTUs higher than 0.1 % in microbial community was consider as the predominant OTUs, and then sorted for comparing the differences among treatment groups. The data of the composition of bacterial community were assessed using each pen as the experimental unit. Growth performance (e.g., Body weight, ADG, ADFI and F/G) and the composition of bacterial community from the phylum to species levels were also tested using one-factor ANOVA program with different treatments as one factor in this study. The data were expressed as the mean ± standard error of the mean (SEM), and significant differences were declared when *P* < 0.05.

## Results

### The effects of different forms EGF-expressed *S. cerevisiae* on the growth performance of weaned piglet

Revealed in the study (Additional file 1: Table S2), at day 14 and 21, respectively, the ADG and ADFI of weaned piglets significantly increased in the INVSc1(EV), INVSc1-EE(+), INVSc1-TE(−), and INVSc1-IE(+) groups when compared with the control group (*P* < 0.05). However, at day 7, 14 and 21, respectively, the ADG, ADFI and F/G showed no marked differences between the INVSc1(EV) strain and other recombinant strains, including INVSc1-EE(+), INVSc1-TE(−), and INVSc1-IE(+) (*P* > 0.10). Furthermore, the effect of T-EGF, EE-EGF, and IE-EGF-expressed *S. cerevisiae* on the growth performance of weaned piglets was further analyzed. The ADG, ADFI and F/G of weaned piglets had no significantly improved in the INVSc1-IE(+) group compared to the INVSc1-TE(−) and INVSc1-EE(+) groups (*P* > 0.10).

### Effectiveness check of the raw reads

Across all samples in the study, 243,598 quality sequences from 3,454,000 reads were classified as bacteria using high-throughput sequencing on PacBio® RS II platform. These sequences were classified to the genus and species level at a 97 % sequence identity threshold. In addition, we also analyzed the percentages of sequences, thus classified along with the nonparametric richness estimates for five groups and for individual animals in each group. The diversities and their abundance statistics were also shown in Additional file 1: Table S3. The average length of quality sequences ranged from 1475.60 bp to 1486.90 bp (1480.35 ± 3.05 bp). Thus it manifested that the average length of full-length 16S rRNA gene was valid assessed among all samples.

To compare the bacterial species richness in all samples, the total number of valid sequences, the number of OTUs, the coverage percentage, and the statistical estimate of the species richness for average 3,041-sequence subsets from each sample at a genetic distance of 3 % were presented in the study. Additionally, there was no significant correlation (Pearson’s correlation, *P* > 0.25) between the number of reads per sample, the number of OTUs, and the estimated number of OTUs.

### The effects of different forms EGF-expressed *S. cerevisiae* on bacterial community of full-length 16S rRNA gene in weaned piglet

#### Population dynamics at species level

**Fig. 1:**
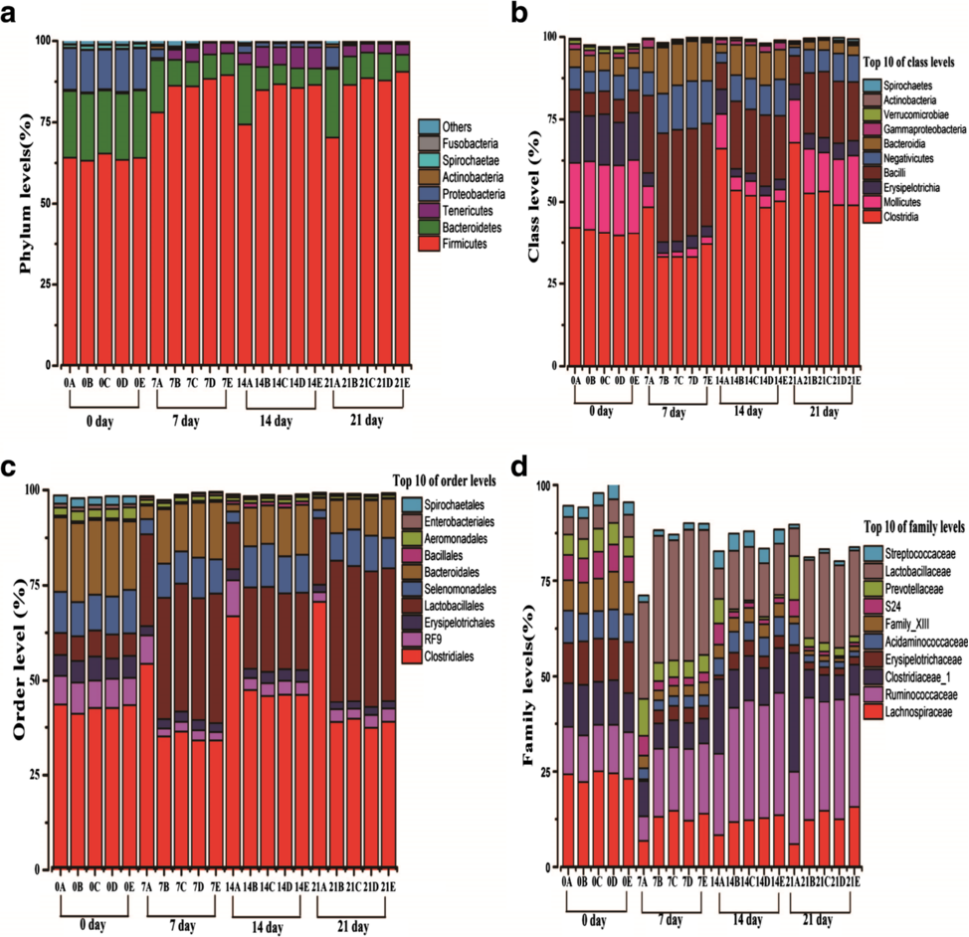
Percentage relative abundance of OTUs observed at the phylum (**a**), class (**b**), order (**c**) and family levels (**d**). Note: 0A, 0B, 0C, 0D, and 0E represent the samples of the control,INVSc1(EV), INVSc1-TE(−), INVSc1-EE(+), and INVSc1-IE(+) groups, respectively, at day 0; 7A, 7B, 7C, 7D, and 7E represent the samples of the control, INVSc1(EV), INVSc1-TE(−), INVSc1-EE(+), and INVSc1-IE(+) groups, respectively, at day 7; 14A, 14B, 14C, 14D, and 14E represent the samples of the control, INVSc1(EV), INVSc1-TE(−), INVSc1-EE(+), and INVSc1-IE(+) groups, respectively, at day 14; 21A, 21B, 21C, 21D, and 21E represent the samples of the control, INVSc1(EV), INVSc1-TE(−), INVSc1-EE(+), and INVSc1-IE(+) groups, respectively, at day 21

At phylum level (Fig. 1a), the similar percentages of the day 0 communities were observed in the control, INVSc1(EV), INVSc1-TE(−), INVSc1-EE(+) and INVSc1-IE(+) groups, respectively. The day 0 communities among all groups were dominated by five most abundant phyla, including

*Firmicutes* (63.15-65.34 %), *Bacteroidetes* (19.49-20.78 %), *Proteobacteria* (12.16-13.08 %), *Spirochaetes* (0.95-1.20 %) and *Tenericutes* (0.32-0.47 %), when compared with the main other phyla.

At day 7, the abundances of *Actinobacteri*a, *Spirochaetae* and *Fusobacteria* were no significant difference among all groups (*P >* 0.05). However, the relative abundances of *Firmicutes* and *Tenericutes* were significantly lower in the control group at 78.05 % and 0.60 % (*P <* 0.01) than those of the INVSc1(EV) group at 86.25 % and 3.11 %, INVSc1-TE(−) group at 86.07 % and 4.31 %, INVSc1-EE(+) group at 88.40 % and 3.69 %, and INVSc1-IE(+) group at 89.54 % and 3.22 %, respectively. In addition, the relative abundances of *Bacteroidetes* (*P <* 0.01) and *Proteobacteria* (*P <* 0.01) in the control group (16.09 % and 0.60 %) were significantly higher than the INVSc1(EV) (7.94 % and 0.54 %), INVSc1-TE(−) (7.51 % and 0.62 %), INVSc1-EE(+) (7.38 % and 0.25 %) and INVSc1-IE(+) (6.59 % and 0.34 %), respectively. Furthermore, the relative abundances of *Firmicutes*, *Tenericutes*, *Bacteroidetes* and *Proteobacteria* had no significant differences between recombinant *S. cerevisiae* groups including the INVSc1(EV), INVSc1-TE(−), INVSc1-EE(+), and INVSc1-IE(+) groups (*P >* 0.05). Apart from the four major phyla, three other phyla were also observed which accounted for only 2.25-2.87 % of the total reads.

At day 14 and 21, the similar trends of the communities were also observed in the control, INVSc1(EV), INVSc1-TE(−), INVSc1-EE(+) and INVSc1-IE(+) groups with the results of day 7.

#### Population dynamics at class level

Revealed in Fig. 1b, at day 0 the six most abundant classes among all groups were the *Clostridia* (39.62-42.10 %), *Mollicutes* (19.68-22.32 %), *Erysipelotrichia* (13.80-15.50 %), *Bacilli* (6.80-7.01 %), *Negativicutes* (6.31-7.30 %), and *Bacteroidia* (4.89-5.31 %), respectively.

At day 7, the levels of *Clostridia* (*P <* 0.01) and *Mollicutes* (*P <* 0.01) at 48.39 % and 6.40 % in the control group were significantly higher than the INVSc1(EV) group at 33.13 % and 1.11 %, INVSc1-TE(−) group at 33.14 % and 1.61 %, INVSc1-EE(+) group at 33.08 % and 1.69 %, and INVSc1-IE(+) group at 37.07 % and 2.22 %, respectively. In contrast, the sequences of *Bacilli* (*P <* 0.01), *Negativicutes* (*P <* 0.01) and *Bacteroidia* (*P <* 0.01) were significantly declined in the control group (23.48 %, 7.08 % and 7.38 %) when compared with the INVSc1(EV) (3.43 %, 33.14 % and 12.01 %), INVSc1-TE(−) (3.07 %, 34.08 % and 13.49 %), INVSc1-EE(+) (3.77 %, 32.63 % and 14.46 %), and INVSc1-IE(+) groups (3.31 %, 31.09 % and 13.00 %), respectively. In terms of the level of *Erysipelotrichia*, there was no significant change among all groups (*P >* 0.05).

At day 14 and 21 day, the similar trends of the six most abundant classes were also observed in the control, INVSc1(EV), INVSc1-TE(−), INVSc1-EE(+) and INVSc1-IE(+) groups with the results of day 7.

In addition, to further assess these six most abundant classes in recombinant *S. cerevisiae* groups, there was no significant change in the INVSc1(EV), INVSc1-TE(−), INVSc1-EE(+) and INVSc1-IE(+) groups at day 7, 14 and 21 day, respectively (*P >* 0.05).

#### Population dynamics at order level

Similar trends were also observed between communities at the order levels (Fig. 1c). At day 0 the six most abundant orders in all groups was the *Clostridiales*, *Bacteroidales*, *Selenomonadales*, *RF9*, *Erysipelotrichales,* and *Lactobacillales*, respectively.

At 7, 14 and 21 day, respectively, the levels of *Clostridiales* (*P <* 0.01) and *RF9* (*P <* 0.05) in the control group were significantly higher than recombinant *S. cerevisiae* groupsincluding the INVSc1(EV), INVSc1-TE(−), INVSc1-EE(+) and INVSc1-IE(+) groups. However, the sequences of *Lactobacillales* (*P <* 0.01), *Selenomonadales* (*P <* 0.05) and *Bacteroidales* (*P <* 0.01) in the control group significantly decreased when compared with those of recombinant *S. cerevisiae* groups. Interestingly, at day 7, 14, and 21 day, respectively, the six most abundant orders (e.g., *Clostridiales*, *Bacteroidales*, *Selenomonadales*, *RF9*, *Erysipelotrichales* and *Lactobacillales*) had no marked change between the INVSc1(EV) strain and other recombinant strains encompassing INVSc1-TE(−), INVSc1-EE(+) and INVSc1-IE(+) groups (*P >* 0.05).

#### Population dynamics at family level

At family level (Fig. 1d), the six families (*Lachnospiraceae*, *Lactobacillaceae*, *Ruminococcaceae*, *Clostridiaceae 1*, *Erysipelotrichaceae*, and *Acidaminococcaceae*) for the *Firmicutes* phylum, and two families (*Prevotellaceae* and *S24*) for the *Bacteriodetes* phylum were more abundant at day 0, respectively.

At day 7, 14 and 21 day, four families (*P <* 0.05) among the *Firmicutes* phylum, *Lachnospiraceae*, *Lactobacillacea*e, *Ruminococcaceae,* and *Erysipelotrichaceae* in the control group were significant lower than those of the INVSc1(EV), INVSc1-TE(−), INVSc1-EE(+) and INVSc1-IE(+) groups. In contrast, *Clostridiaceae 1*(*P <* 0.01), which also belongs to the *Firmicutes* phylum, significantly increased in the control group when compared with the INVSc1(EV), INVSc1-TE(−), INVSc1-EE(+) and INVSc1-IE(+) groups. The *Acidaminococcaceae* family, from the *Firmicutes* phylum, remained unchanged in all groups (*P >* 0.05). In the meanwhile, the *Prevotellaceae* and *S24* family, from the *Bacteriodetes* phylum, also were significant higher in the control group than those of the INVSc1(EV), INVSc1-TE(−), INVSc1-EE(+) and INVSc1-IE(+) groups (*P <* 0.01).

In addition, at day 7, 14 and 21, respectively, the relative abundance of seven families, including *Lachnospiraceae*, *Lactobacillaceae, Ruminococcaceae*, *Clostridiaceae 1*, *Erysipelotrichaceae*, *Prevotellaceae* and *S24*, had no significantly change among recombinant *S. cerevisiae* groups encompassing the INVSc1(EV), INVSc1-TE(−), INVSc1-EE(+) and INVSc1-IE(+) groups (*P >* 0.05).

#### Population dynamics at genus level

**Fig. 2:**
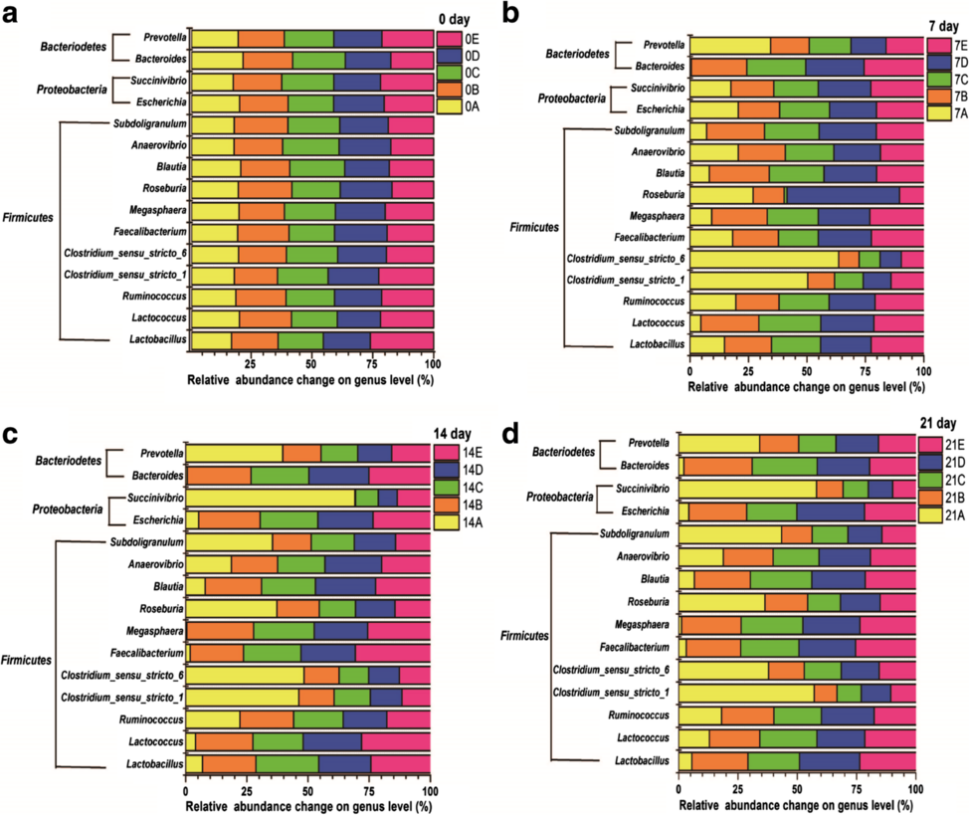
Percentage of relative abundance change at genus level. Note: (**a**) 0A, 0B, 0C, 0D, and 0E represent the samples of the control, INVSc1(EV), INVSc1-TE(−), INVSc1-EE(+), and INVSc1-IE(+) groups, respectively, at day 0; (**b**) 7A, 7B, 7C, 7D, and 7E represent the samples of the control, INVSc1(EV), INVSc1-TE(−), INVSc1-EE(+), and INVSc1-IE(+) groups, respectively, at day 7; (**c**) 14A, 14B, 14C, 14D, and 14E represent the samples of the control, INVSc1(EV), INVSc1-TE(−), INVSc1-EE(+), and INVSc1-IE(+) groups, respectively, at day 14; (**d**) 21A, 21B, 21C, 21D, and 21E represent the samples of the control, INVSc1(EV), INVSc1-TE(−), INVSc1-EE(+), and INVSc1-IE(+) groups, respectively, at day 21

As shown in the Fig. 2, a 97 % identity level was applied to classify the sequences at genus level. 53-56 %, 52-55 %, 50-53 % and 49-53 % of the total number of sequences were taxonomically assigned at day 0, 7, 14 and 21 all groups, respectively. There was no consistency observed at genus level in any of all groups.

At day 0 all groups (Fig. 2a), *Lactobacillus* (4.63-5.55 %) and *Clostridium* genera (6.69-7.28 %) from the *Firmicutes* phylum were most abundant, followed by *Escherichia* (3.72-4.13 %) and *Succinivibrio* (4.50-5.12 %) genera from the *Proteobacteria* phylum, *Blautia* genera (3.20-3.97 %) from the *Firmicutes* phylum, *Bacteroides* (1.82-2.24 %) and *Prevotella* (1.76-1.97 %) genera from the *Bacteriodetes* phylum.

To get a clearer understanding of the variation among all groups at day 7, 14 and 21, respectively, the relative abundance was plotted for the genera that had significant difference (*P <* 0.01) (Fig. 2b, c and d). The members of *Lactobacillus*, *Blautia* and *Bacteroides* significantly decreased in the control group when compared with the INVSc1(EV), INVSc1-TE(−), INVSc1-EE(+) and INVSc1-IE(+) groups (*P <* 0.01), whereas the sequences of *Clostridium* and *Prevotella* appeared the contrary results (*P <* 0.01). The relative abundances of *Succinivibrio*, *Subdoligranulum* and *Ruminococcus* remained unchanged between the control group and recombinant *S. cerevisiae* groups encompassing the INVSc1(EV), INVSc1-TE(−), INVSc1-EE(+) and INVSc1-IE(+) groups (*P >* 0.05).

Furthermore, except for the *Succinivibrio* genus (*P <* 0.01), the relative abundances of six genera members encompassing *Lactobacillus*, *Clostridium*, *Subdoligranulum*, *Escherichia*, *Succinivibrio*, *Bacteroides* and *Prevotella* had no significant difference between the INVSc1(EV) group and other recombinant strains including INVSc1-TE(−), INVSc1-EE(+) and INVSc1-IE(+) (*P >* 0.05).

#### Population dynamics at species level

At 99 % similarity level, 49.26-53.75 %, 47.22-50.31 %, 45.06-49.82 % and 48.77-50.81 % of day 0, 7, 14 and 21 all group sequences were classified to known cultured species. The ten most abundant OTUs of samples in all groups encompassing the control, INVSc1(EV), INVSc1-TE(−), INVSc1-EE(+), and INVSc1-IE(+) groups from 0 day to 21 day were shown in Additional file 1: Table S4-S7.

**Fig. 3:**
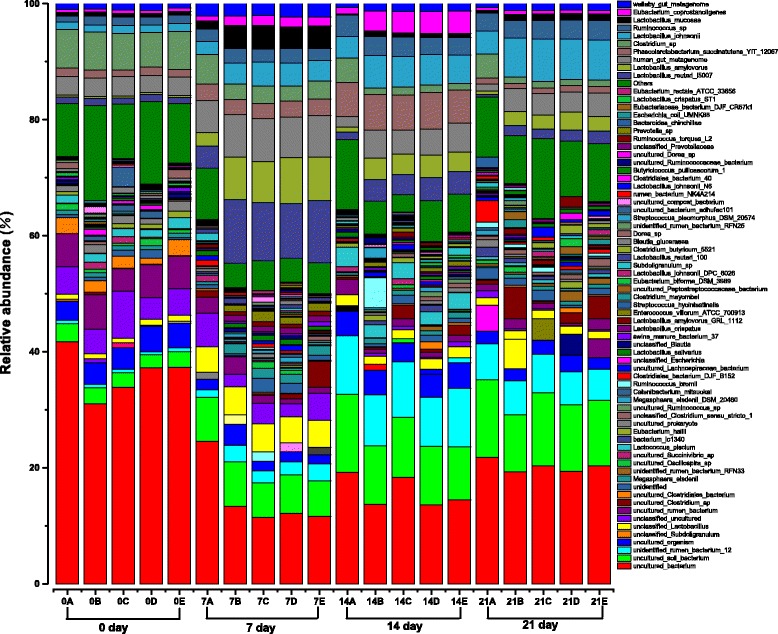
Bacterial community at the species level. Note: 0A, 0B, 0C, 0D, and 0E represent the samples of the control, INVSc1(EV), INVSc1-TE(−), INVSc1-EE(+), and INVSc1-IE(+) groups, respectively, at day 0; 7A, 7B, 7C, 7D, and 7E represent the samples of the control, INVSc1(EV), INVSc1-TE(−), INVSc1-EE(+), and INVSc1-IE(+) groups, respectively, at day 7; 14A, 14B, 14C, 14D, and 14E represent the samples of the control, INVSc1(EV), INVSc1-TE(−), INVSc1-EE(+), and INVSc1-IE(+) groups, respectively, at day 14; 21A, 21B, 21C, 21D, and 21E represent the samples of the control, INVSc1(EV), INVSc1-TE(−), INVSc1-EE(+), and INVSc1-IE(+) groups, respectively, at day 21

**Fig. 4:**
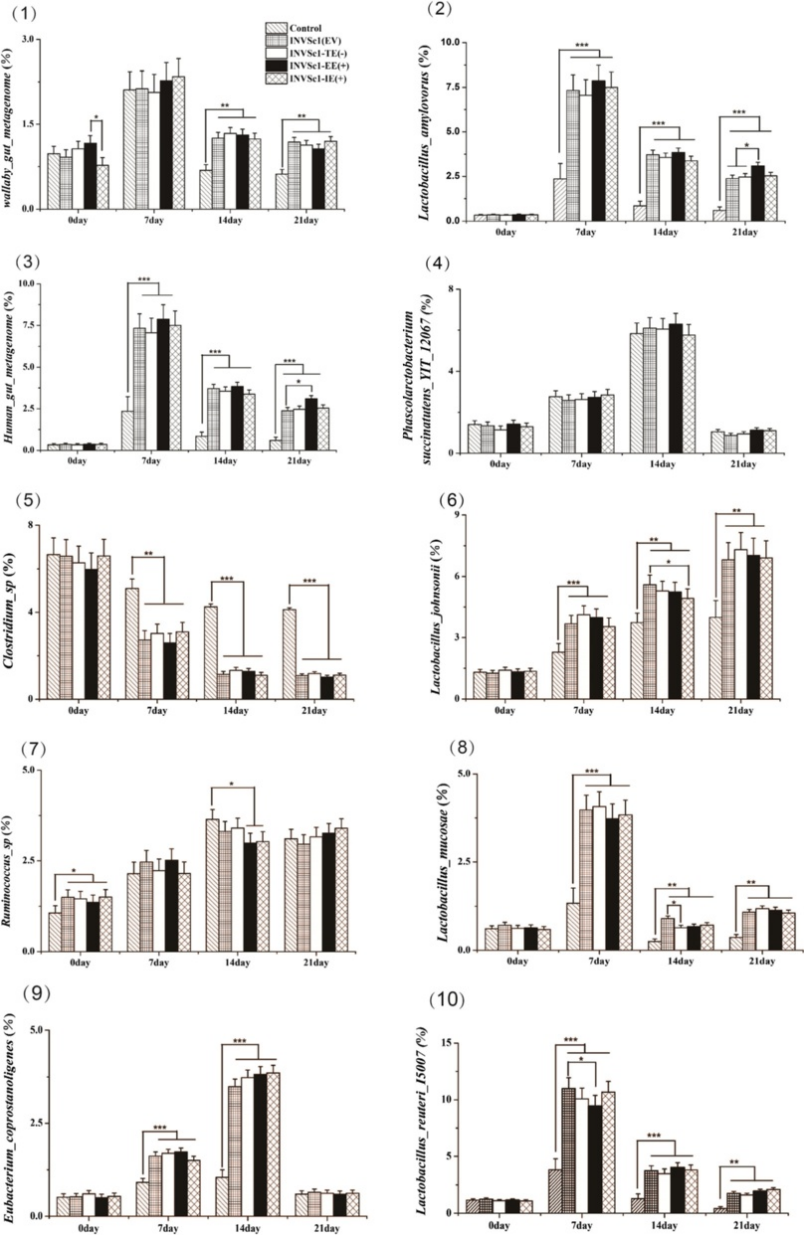
Top 10 relative abundances belonging to bacterial species at day 0, 7, 14 and 21. Note: *significant at the 5.00 % level;**significant at the 1.00 % level;***significant at the 0.10 % level. Error bars represent standard deviation from the mean

Revealed in Figs. 3 and 4, at day 0, the ten most abundant OTUs remained unchanged among all groups. With regard to *Lactobacillus*, the sequences assigned to species level belonged to *L. johnsonii* (OTU61), *L. reuteri* (OUT178), *L. mucosae* (OUT144) and *L. amylovorus* (OUT52), respectively. At day 7, 14 and 21, respectively, *L. johnsonii*, *L. reuteri*, *L. mucosae* and *L. amylovorus* were presented at the lower numbers in the control group when compared with recombinant *S. cerevisiae* groups encompassing INVSc1(EV), INVSc1-TE(−), INVSc1-EE(+), and INVSc1-IE(+) (*P <* 0.05). The relative abundance changes in regard to the enteric pathogens, such as *Clostridium sp* (OUT174), in the control group was higher than those of the INVSc1(EV), INVSc1-TE(−), INVSc1-EE(+), and INVSc1-IE(+) groups at day 7, 14 and 21, respectively (*P <* 0.05). The species levels of *Human gut metagenome* (OTU37), *Wallaby gut metagenome* (OTU1469)*,* and *Eubacterium coprostanoligenes* (OTU357) were significant lower in the control group than those of the INVSc1(EV), INVSc1-TE(−), INVSc1-EE(+), and INVSc1-IE(+) groups at day 7, 14 and 21 (*P <* 0.05), respectively. In addition, at day 7, 14 and 21, respectively, the relative abundances of *Phascolarcto bacterium succinatutens YIT 12067* (OTU118) and *Ruminococcus sp* (OTU298) had no significant difference in the control group compared to the INVSc1(EV), INVSc1-TE(−), INVSc1-EE(+), and INVSc1-IE(+) groups (*P >* 0.05).

To analyze a clearer understanding of the variation of the ten most abundant OTUs among recombinant *S. cerevisiae* groups including the INVSc1(EV), INVSc1-TE(−), INVSc1-EE(+), and INVSc1-IE(+) groups, there were no significant difference among them at day 7, 14 and 21, respectively (*P >* 0.05).

### Clustering analysis of bacterial community

**Fig. 5:**
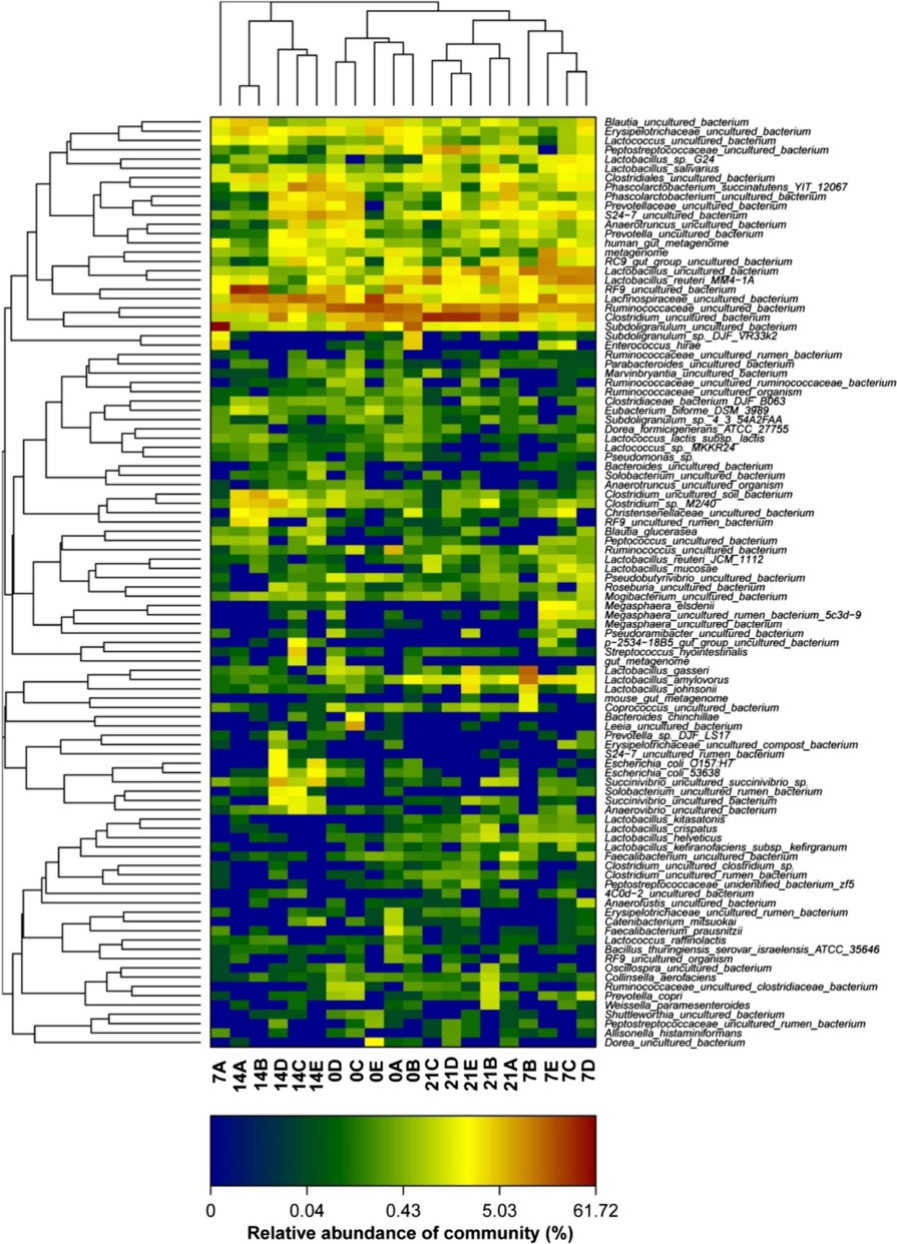
Bacterial distribution of heatmap at the species level. Double dendrogram showing the bacterial distribution (species level) among the all samples. The bacterial phylogenetic tree was calculated using the neighbor-joining method, and the relationship among samples was determined using Bray distance and the complete clustering method. Total 100 species with the abundance higher than 0.1 % within total bacteria were sorted for the analysis. The heatmap plot depicts the relative percentage of each bacterial species (variables clustering on the Y-axis) within each sample (X-axis clustering). The relative values for the bacterial species are depicted by color intensity in the legend indicated at the top of the figure. Clusters based on the distance of the different samples along the X-axis and the bacterial species along the Y-axis are indicated at the top and bottom of the figure, respectively. Note: 0A, 0B, 0C, 0D, and 0E represent the samples of the control, INVSc1(EV), INVSc1-TE(−), INVSc1-EE(+), and INVSc1-IE(+) groups, respectively, at day 0; 7A, 7B, 7C, 7D, and 7E represent the samples of the control, INVSc1(EV), INVSc1-TE(−), INVSc1-EE(+), and INVSc1-IE(+) groups, respectively, at day 7; 14A, 14B, 14C, 14D, and 14E represent the samples of the control, INVSc1(EV), INVSc1-TE(−), INVSc1-EE(+), and INVSc1-IE(+) groups, respectively, at day 14; 21A, 21B, 21C, 21D, and 21E represent the samples of the control, INVSc1(EV), INVSc1-TE(−), INVSc1-EE(+), and INVSc1-IE(+) groups, respectively, at day 21

Based on the heatmap analysis of microbial community on species level (Fig. 5), at day 0, the bacterial community profiles disclosed that the samples among all groups encompassing the control, INVSc1(EV), INVSc1-TE(−), INVSc1-EE(+), and INVSc1-IE(+) were grouped together with a high similarity. Thus, it manifested that bacterial communities in the samples of different groups at the initial stage included mostly similar sequences.

Furthermore, the bacterial community profiles at species level had showed that samples in each treatment group at day 7, 14 and 21 were grouped together with a low similarity. When viewed in this way, the bacterial community of samples in each treatment group had significant change with different growth stages. Interestingly, at each growth stage, the bacterial community profiles of samples in recombinant *S. cerevisiae* groups, including INVSc1(EV), INVSc1-TE(−), INVSc1-EE(+), and INVSc1-IE(+), were grouped together with a high similarity, while the control group was outlier from recombinant *S. cerevisiae* groups.

## Discussion

During and shortly weaning, some approaches to improve microbial community of the GIT in mammals are still explored in recent years [24–26]. Interestingly, the combination of some growth factors (e.g., EGF, insulin, glucagon-like peptide-2, and insulin-like growth factor) and probiotics (e.g., *Lactobacillu*, *S.cerevisiae*, and *Bifidobacterium bifidum*) has piqued the great interest of the researchers focused on solving the gastrointestinal dysfunction in human, piglet, and other mammals [10, 11, 19]. In fact, there exists only limited research to consider the application of the approach.

In the current research, we had evaluated the effects of dietary supplementation of T-EGF, EE-EGF, and IE-EGF-expressed *S. cerevisiae* on bacterial community of full-length 16S rRNA in duodenum. At day 7, 14 and 21, respectively, we had found that recombinant *S. cerevisiae* groups, including the INVSc1(EV), INVSc1-TE(−), INVSc1-EE(+), and INVSc1-IE(+), had discernable influences on the host physiology as inferred from bacterial community when compared with those of the control group. In fact, the application of *S.cerevisiae* and its products as probiotics had been well-documented in animal models or humans [27–30]. Similarly feeding the live *yeast* cells, such as *S. cerevisiae*, did significant affect bacterial communities and performance growth of the piglets [31, 32].

In the study, the effects of recombinant *S. cerevisiae* on four major phyla: *Firmicutes*, *Bacteroidetes*, *Proteobacteria* and *Tenericutes* in duodenum were analyzed. The percentage of *Firmicutes,* and *Tenericutes* significantly increased in recombinant *S. cerevisiae* groups, including the INVSc1(EV), INVSc1-TE(−), INVSc1-EE(+), and INVSc1-IE(+), when compared with the control group. Additionally, the population of enteric pathogens, such as *Clostridium* and *Prevotella*, significantly decreased in these recombinant *S. cerevisiae* groups. Furthermore, at genus level, the count of *Lactobacilli* was also higher in recombinant *S. cerevisiae* groups than those of the control group. The outer layer of *S. cerevisiae* was composed of the mannose associated protein, which called the mannan and mannose oligosaccharide that might function as prebiotic components [31]. In the humanized microbiome mouse models, it had also shown that the mannose oligosaccharide could stimulate an alteration in the counts of *lactobacilli* via facilitating an increase in *bifidobacteria* [33]. In addition, the diet or drinking water supplemented with the inulin-type fructans resulted in less diarrhoeal occurrence, and reduced pathogen shedding in animals [34]. Thus, adding recombinant *S. cerevisiae* into the diet of animal was an ideal approach to improve the development and microbial community of the GIT while exogenously supplementing probiotics.

Furthermore, we also further compared the proportions of bacterial communities between T-EGF, EE-EGF and IE-EGF at day 7, 14 and 21, respectively. Revealed in this study, the proportions of bacterial communities from the phylum to species levels remained unchanged among them at day 7, 14 and 21, respectively. In the meanwhile, to get a clearer understanding of the variation of the bacterial community in these recombinant *S. cerevisiae* including the INVSc1-TE(−), INVSc1-EE(+), and INVSc1-IE(+), there was no significant change in the proportion of bacterial communities observed at all OTU levels among them at day 7, 14 and 21, respectively. Our current results were no consistent with previously hypothesis that the biological activities of IE-EGF were better than either EE-EGF or T-EGF in weaned rats and piglets [11, 19]. Up to now, little was known regarding exogenous EGF to elicit the effects on microbial community of suckling neonates or weaned mammals. In fact, EGF receptors had been suggested to be 10 times more prevalent than EGF itself in the intestine, indicating that an exogenous supplement of EGF might be effectively taken up by developing animals [35]. In our previous studies [36], we had indicated that the diet supplemented with different expression forms EGF-expressed *S. cerevisiae* could stimulate the mRNA expression of digestive enzymes and EGF-R, and increase its enzyme activities to facilitate the development and immune function of the duodenum in weaned piglets. Herein, an exogenous EGF was effective in the duodenum. However, revealed in this study, there was no significant difference associated with duodenal microbiotas of weaned piglet between recombinant EGF-expressed *S. cerevisiae* and empty vector-expressed *S. cerevisiae*. When viewed in this way, in this paper, altered the microbiome effect was really *S. cerevisiae*, and then different forms of recombinant EGF, including T-EGF, EE-EGF and IE-EGF, did not appear to make a significant difference to the microbiome of weaned piglet.

To understand and exploit the impact of exogenous recombinant *S. cerevisiae* on the GIT of weaned piglets, the content, diversity and functioning of microbial community of full-length 16S rRNA gene were deciphered using high-throughput sequencing on PacBio® RS II platform. Bacterial 16S rRNA gene contained “hypervariable regions” (V1-V9) that demonstrated considerable sequence diversity among different bacteria [37]. Unfortunately, hypervariable regions in 16S rRNA gene exhibited different degrees of sequence diversity, and no single hypervariable region was able to distinguish all bacteria [38]. It manifested that only complete 16S rRNA sequence rather than one of hypervariable regions (V1-V9) gave an accurate measure of taxonomic diversity [23, 39]. Therefore, systematic studies that assessed the relative advantage of the complete 16S rRNA sequence for specific diagnostic goal were needed. Indeed, the average length of full-length 16S rRNA gene was validly assessed in all samples in the current research and other new research.

Besides, although diversity estimates based on the OTUs did differ amongst the individual piglets, significant perturbations were observed in the GIT microbiota of piglet according to different dietary treatments. Each individual piglet was evaluated using the heatmap clustering, and then were clustered based on their dietary treatments. Thus, the sequence analysis supported the dietary pattern at day 7, 14 and 21, respectively. The 16S rRNA sequence-based comparisons of human [21, 40], swine [41] and canine [42] faecal microbiota had also revealed the high levels of inter-individual variations. In the meanwhile, the Shannon indices, which obtained for these piglets with the normalized reads, showed a substantial increase in the sequencing tags leading to the higher diversity indices. However, the microbial composition also varied in each individual [43], and depended on the host response to that given different diets. Adding the *S. cerevisiae* to the diets could potentially alter the GIT microbiota of piglet, however such changes depended on its endogenous microbiota which caused a divergence in relative response to that given different diets [31].

In addition, after analyzed effectiveness of the raw reads, the average coverages of sequences from all samples was 80.85 %(73.00-95.00 %), and the individual rarefaction curves of all samples did not level off at the sequencing depth of 6,000 (Additional file 1: Figure S1). Therefore, this sequencing depth in the current research was not enough to cover the whole bacterial diversity. Actually, the analysis of microbial community was only as good as the underlying biological question, study design, DNA extraction method, PCR conditions, sequencing, and bioinformatic analysis. Meanwhile, the presence of chimeric 16S rRNA gene sequences and its sequencing errors had an important influence on the biodiversity reflected in microbial ecology studies [44]. To correct for these sources of error previous evidence had proposed many solutions, which could be tailored to a particular analysis [44, 45]. The rate of chimerism, drift and sequencing error rate in the representation of community structure was also calculated using the inclusion of a mock community sample on each sequencing run. Thus, tailoring the genetic diversity and richness of mock community to the samples would be a useful resource for the microbial ecology research. Similarly, re-sequencing a control sample on each sequencing run was a useful datum to include if a control sample was representative of the biodiversity and complexity of the other samples being analyzed. In addition, it was likely that insidious variation in thermalcycler calibration, reagent concentrations, and other factors were the cause of this technical variation [44]. Thus, it deserves further sequencing to detect more bacterial species in the samples of piglets in the future.

In fact, more robust studies still are required with more animals, fatty acid estimation of intestine contents and their bacterial community proportions of the individual subjects, which can accurately reveal the interactions between the different diets and bacterial communities. Thus, such studies will reveal the inter-play among diet-mediated alterations in bacterial secondary metabolites and their symbiotic relationships, which can make such studies more meaningful and therefore contribute to the development of new health related nutrition strategies.

## Conclusion

In conclusion, the current research suggested that recombinant *S. cerevisiae* had the potential to be used as a supplement for increasing potentially probiotic (e.g., *Lactobacilli* and *Lactococcus*), and reducing potentially pathogenic bacteria (e.g., *Clostridium* and *Prevotella*) in weaned piglet after weaning. However, the populations of potentially enterobacterial and probiotic from the phylum to species levels remained unchanged among different forms EGF-expressed *S. cerevisiae*, including the INVSc1-TE(−), INVSc1-EE(+), and INVSc1-IE(+) strains. Hence, adding endogenous EGF to the diets of weaned piglets had no influence on the bacterial community of the duodenum, but endogenous *S. cerevisiae* had the potential to serve as dietary supplement in animal nutrition to improve the bacterial composition and to reduce the potential for diarrhea diseases.

## Abbreviations

EE-EGF, extracellular expressing-EGF; IE-EGF*,* intracellular expressing-EGF; INVSc1-EE(+), EE-EGF expressing-*Saccharomyces cerevisiae*; INVSc1-IE(+), IE-EGF expressing-*Saccharomyces cerevisiae*; INVSc1-TE(−), T-EGF protein expressing-*Saccharomyces cerevisiae*; T-EGF, tagged-EGF (carry the protein tag from the expression vector)

## Additional file

Additional file 1: MCRO-D-16-00176R1 Supplemental data Zhang *et al*. The supplemental data consist of seven tables and one figure. **Table S1**. Composition and nutrient levels of the basal diet. **Table S2**. The effects of different forms EGF-expressing *S. cerevisiae* on the growth performance of weaned piglet. **Table S3**. Sequences from the samples of all groups at day 0, 7, 14 and 21, respectively. **Table S4**. The percentages of top 10 relative abundances at species level at day 0. **Table S5**. The percentages of top 10 relative abundances at species level at day 7. **Table S6**. The percentages of top 10 relative abundances at species level at day 14. **Table S7**. The percentages of top 10 relative abundances at species level at day 21. **Figure S1**. Rarefaction curves for the animals at day 0, 7, 14 and 21. Note: A = Control group; B = INVSc1(EV) group; C = INVSc1-TE(−) group; D = INVSc1-EE(+) group; E = INVSc1-IE(+) group. (DOCX 1495 kb)
