# Erratum to: Analysis of the duodenal microbiotas of weaned piglet fed with epidermal growth factor-expressed *Saccharomyces cerevisiae*

**DOI:** 10.1186/s12866-016-0874-5

**Published:** 2016-10-31

**Authors:** Zhongwei Zhang, Lili Cao, Yan Zhou, Shujin Wang, Lin Zhou

**Affiliations:** 1 Department of Intensive Care Unit, West China Hospital, Sichuan University, Chengdu, Sichuan 610041 People’s Republic of China; 2 Medical School, Chengdu University, Chengdu, Sichuan 610041 People’s Republic of China; 3 Shenzhen Premix Inve Nutrition Co., LTD, Shenzhen, 518103 People’s Republic of China; 4 Human and Animal Physiology, Wageningen University, De Elst 1, 6708 WD Wageningen, The Netherlands

## Erratum

Unfortunately, incorrect author affiliations were presented in the original version of this article [1]. These affiliations have now been corrected within this erratum.

An updated contact address for Shujin Wang has also been provided:

Human and Animal Physiology, Wageningen University, De Elst 1, 6708 WD Wageningen, The Netherlands.
