# Supplementary material for: Analysis of the duodenal microbiotas of weaned piglet fed with epidermal growth factor-expressed Saccharomyces cerevisiae
Source: BMC Microbiol. 2016 Jul 28;16:166. doi: 10.1186/s12866-016-0783-7 (PMC4964059; doi:10.1186/s12866-016-0783-7)
Supplement: Additional file 1: — MCRO-D-16-00176R1 Supplemental data Zhang et al. The supplemental data consist of seven tables and one figure. Table S1. Composition and nutrient levels of the basal diet. Table S2. The effects of different forms EGF-expressing S. cerevisiae on the growth performance of weaned piglet. Table S3. Sequences from the samples of all groups at day 0, 7, 14 and 21, respectively. Table S4. The percentages of top 10 relative abundances at species level at day 0. Table S5. The percentages of top 10 relative abundances at species level at day 7. Table S6. The percentages of top 10 relative abundances at species level at day 14. Table S7. The percentages of top 10 relative abundances at species level at day 21. Figure S1. Rarefaction curves for the animals at day 0, 7, 14 and 21. Note: A = Control group; B = INVSc1(EV) group; C = INVSc1-TE(−) group; D = INVSc1-EE(+) group; E = INVSc1-IE(+) group. (DOCX 1495 kb) [file 12866_2016_783_MOESM1_ESM.docx]

Supplementary Information

**Analysis of the duodenal microbiotas of weaned-piglet fed with epidermal growth factor-expressing Saccharomyces cerevisiae**

*Zhongwei Zhang^1^*, *Lili Cao^4^*, *Yan Zhou^1^*, *Theo GMF Gorgels^2^*, *Tos TJM Berendschot^2^*, *Shujin Wang^2*^ and Lin Zhou^3*^*

*1. Department of Intensive Care Unit, West China Hospital, Sichuan University, Chengdu, 610041, Sichuan, P. R. China*

*2. Human and Animal Physiology, Wageningen University, 6700 AH, Wageningen, The Netherlands*

*3. Shenzhen Premix Inve Nutrition Co., LTD, Shenzhen, 518103, PR China*

*4. Medical School, Chengdu University, Chengdu, 610041, Sichuan, P. R. China*

**. To whom correspondence should be addressed. Shujin Wang and Lin Zhou equally contributed to this work and should be considered as co-corresponding authors.*

**** Author for correspondence:***

Dr. Shujin Wang

Tel: +31 685417064

E-mail address: [wangrui19880622@sina.com](mailto:wangrui19880622@sina.com) (Shujin Wang, corresponding author)

[zhouwei382@163.com](mailto:zhouwei382@163.com) (Lin Zhou, co-corresponding author)

[zhangzhongwei1@medmail.com.cn](mailto:zhangzhongwei1@medmail.com.cn) (Zhongwei Zhang, first author)

[993077411@qq.com](mailto:993077411@qq.com) (Lili Cao, second author)

[zhouyanhxicu@163.com](mailto:zhouyanhxicu@163.com)(Zhongwei Zhang, third author)

**Table S1. Composition and nutrient levels of the basal diet (as fed-basis).**

| Item | Content, % |
| --- | --- |
| Ingredients |  |
| Corn (CP^4^ 8.70%) | 55.94 |
| Wheat | 5.50 |
| Whey, dried | 5.70 |
| Soybean meal | 10.82 |
| Soybean protein (CP 65.00%) | 12.65 |
| Soybean oil | 1.60 |
| Fishmeal | 3.50 |
| L-Lysine-HCl | 0.12 |
| DL-Methionine | 0.11 |
| L-Threonine | 0.05 |
| Sugar | 1.50 |
| Limestone | 0.80 |
| Salt | 0.26 |
| Dicalcium phosphate | 0.55 |
| Vitamin premix^1^ | 0.40 |
| Trace mineral premix^2^ | 0.60 |
| Total | 100.00 |
| Energy and nutrient composition^3^ |  |
| ME^5^, kcal/kg | 3,420 |
| CP^4^, % | 22.80 |
| Crude fat, % | 4.40 |
| Lys, % | 1.42 |
| Met, % | 0.52 |
| Met + Cys, % | 0.85 |

^1^Vitamins were provided in the following amounts per kilogram of the diet: vitamin A, 10,000 IU; vitamin D_3_, 1,500 IU; vitamin E, 50 IU; vitamin K_3_, 2.50 mg; vitamin B_12_, 60 μg; vitamin B_1_, 4.50 mg; vitamin B_2_, 12 mg; niacin, 60 mg; pantothenic acid, 36 mg; folic acid, 1 mg; vitamin B_6_, 10 mg; biotin, 0.50 mg; and vitamin C, 200 mg.

^2^Trace minerals were provided in the following amounts per kilogram of the diet: Fe, 100 mg; Cu, 6 mg; Mn, 4 mg; Zn, 100 mg; I, 0.30 mg; Co, 0.14 mg; and Se, 0.30 mg.

^3^Calculated values unless indicated otherwise.

^4^CP=Crude protein.

^5^ME= Metabolizable energy.

**Table S2. The effects of different forms EGF-expressing *S. cerevisiae* on the growth performance of weaned piglet^1^.**

|  | Control | INVSc1(EV) | INVSc1-TE(+) | INVSc1-EE(+) | INVSc1-IE(+) |  |  |
| --- | --- | --- | --- | --- | --- | --- | --- |
| Items | Means | | | | | SEM | *P*-values |
| I-BW (kg) | 6.10 | 6.09 | 6.10 | 6.10 | 6.09 | 0.03 | ns |
| F-BW (kg) | 11.50^c^ | 12.17^bc^ | 12.48^ab^ | 12.90^ab^ | 13.17^a^ | 0.24 | ** |
| ADG (g) |  |  |  |  |  |  |  |
| 0-7d | 209.29 | 207.74 | 224.70 | 232.14 | 232.50 | 0.43 | ns |
| 0-14d | 247.35^b^ | 246.16^b^ | 250.30^b^ | 291.96^a^ | 255.39^ab^ | 0.79 | * |
| 0-21d | 257.26^c^ | 289.29^b^ | 304.01^ab^ | 323.59^ab^ | 337.14^a^ | 11.55 | *** |
| ADFI (g) |  |  |  |  |  |  |  |
| 0-7d | 279.97 | 305.36 | 276.59 | 276.12 | 296.30 | 0.60 | ns |
| 0-14d | 369.36^bc^ | 409.75^a^ | 363.63^c^ | 394.92^ab^ | 401.98^a^ | 1.13 | ** |
| 0-21d | 439.52^b^ | 481.76^a^ | 437.58^b^ | 462.11^ab^ | 471.01^a^ | 8.47 | ** |
| F/G |  |  |  |  |  |  |  |
| 0-7d | 1.34 | 1.47 | 1.23 | 1.19 | 1.27 | 0.79 | ns |
| 0-14d | 1.49 | 1.66 | 1.45 | 1.35 | 1.57 | 0.81 | ns |
| 0-21d | 1.71^a^ | 1.67^a^ | 1.44^b^ | 1.43^b^ | 1.40^b^ | 0.04 | * |

**^1^Note:** I-BW= initial body weight; F-BW= final body weight; ADFI= average daily feed intake; ADG = average daily gain; F/G = feed to gain ratio. ^a,b,c,^ Means within the same row without common superscripts differ significantly(*P*<0.05), ns at *P*>0.05; *significant at the 5.00% level;**significant at the 1.00% level;***significant at the 0.10% level.

**Table S3****. Sequences from the samples of all groups at day 0, 7, 14 and 21, respectively^1^.**

| Sample ID | 3﹪distance | | | | | | |
| --- | --- | --- | --- | --- | --- | --- | --- |
|  | Valid Sequences | Average-Length  (bp) | OTU | Chao | Shannon | Simpson | Coverage |
| 0A | 5,248 | 1,486.90 | 1,359 | 2,914.30 | 5.18 | 0.02 | 0.89 |
| 0B | 4,300 | 1,479.00 | 1,567 | 6,167.00 | 6.04 | 0.01 | 0.80 |
| 0C | 3,800 | 1,480.10 | 853 | 6,874.19 | 6.21 | 0.01 | 0.73 |
| 0D | 3,775 | 1,482.50 | 1,211 | 9,939.77 | 6.33 | 0.01 | 0.73 |
| 0E | 4,000 | 1,482.20 | 1,056 | 5,334.12 | 5.58 | 0.02 | 0.81 |
| 7A | 5,050 | 1,481.80 | 1,186 | 6,657.04 | 6.06 | 0.01 | 0.76 |
| 7B | 4,106 | 1,480.90 | 1,096 | 5,055.05 | 5.99 | 0.01 | 0.74 |
| 7C | 3,800 | 1,485.10 | 1,300 | 4,222.28 | 6.03 | 0.01 | 0.80 |
| 7D | 3,600 | 1,483.90 | 1,211 | 4,437.53 | 5.90 | 0.01 | 0.82 |
| 7E | 5,400 | 1,479.80 | 921 | 4,201.63 | 5.02 | 0.03 | 0.85 |
| 14A | 5,200 | 1,476.70 | 1,340 | 1,699.89 | 4.72 | 0.04 | 0.87 |
| 14B | 4,049 | 1,483.20 | 1,328 | 3,180.77 | 5.49 | 0.01 | 0.88 |
| 14C | 3,344 | 1,478.90 | 789 | 7,499.67 | 6.18 | 0.01 | 0.76 |
| 14D | 3,700 | 1,479.20 | 824 | 8,081.60 | 6.38 | 0.00 | 0.73 |
| 14E | 4,725 | 1,477.40 | 1,388 | 7,857.32 | 5.88 | 0.01 | 0.81 |
| 21A | 4,464 | 1,478.20 | 1,047 | 8,947.78 | 6.45 | 0.00 | 0.74 |
| 21B | 4,049 | 1,475.60 | 1,116 | 7,024.93 | 6.08 | 0.01 | 0.75 |
| 21C | 4,240 | 1,481.60 | 1,479 | 1,127.57 | 5.53 | 0.04 | 0.95 |
| 21D | 3,900 | 1,478.70 | 1,431 | 7,226.51 | 5.53 | 0.02 | 0.80 |
| 21E | 5,600 | 1,475.80 | 1,088 | 1,182.1 | 5.72 | 0.02 | 0.95 |

**^1^Note:** 0A, 0B, 0C, 0D, and 0E represent the samples of the control, INVSc1(EV), INVSc1-TE(-), INVSc1-EE(+), and INVSc1-IE(+) groups, respectively, at day 0;

7A, 7B, 7C, 7D, and 7E represent the samples of the control, INVSc1(EV), INVSc1-TE(-), INVSc1-EE(+), and INVSc1-IE(+) groups, respectively, at day 7;

14A, 14B, 14C, 14D, and 14E represent the samples of the control, INVSc1(EV), INVSc1-TE(-), INVSc1-EE(+), and INVSc1-IE(+) groups, respectively, at day 14;

21A, 21B, 21C, 21D, and 21E represent the samples of the control, INVSc1(EV), INVSc1-TE(-), INVSc1-EE(+), and INVSc1-IE(+) groups, respectively, at day 21.

**Table S4. The percentages of top 10 relative abundances at species level at day 0.**

|  | 0 day | | | | |  |  |
| --- | --- | --- | --- | --- | --- | --- | --- |
|  | 0A | 0B | 0C | 0D | 0E |  |  |
|  |  |  |  |  |  |  |  |
|  | Means of mixed samples | | | | | SEM | *P*-values |
| *Lactobacillus_reuteri_I5007* | 1.160 | 1.249 | 1.098 | 1.163 | 1.087 | 0.106 | ns |
| *Lactobacillus_amylovorus* | 0.336 | 0.352 | 0.330 | 0.360 | 0.355 | 0.041 | ns |
| *human_gut_metagenome* | 3.210 | 3.180 | 3.327 | 2.944 | 3.162 | 0.439 | ns |
| *Phascolarctobacterium_succinatutens_YIT_12067* | 1.404 | 1.340 | 1.141 | 1.427 | 1.294 | 0.185 | ns |
| *Clostridium_sp* | 6.654 | 6.577 | 6.277 | 5.966 | 6.590 | 0.763 | ns |
| *Lactobacillus_johnsonii* | 1.305 | 1.256 | 1.410 | 1.331 | 1.355 | 0.142 | ns |
| *Ruminococcus_sp* | 1.068^b^ | 1.499^a^ | 1.455^a^ | 1.359^a^ | 1.507^a^ | 0.199 | * |
| *Lactobacillus_mucosae* | 0.611 | 0.709 | 0.623 | 0.636 | 0.590 | 0.085 | ns |
| *Eubacterium_coprostanoligenes* | 0.515 | 0.522 | 0.603 | 0.502 | 0.536 | 0.011 | ns |
| *wallaby_gut_metagenome* | 0.977^a^ | 0.916^a^ | 1.065^a^ | 1.166^a^ | 0.775^b^ | 0.132 | * |

**^1^Note:** 0A, 0B, 0C, 0D, and 0E represent the samples of the control, INVSc1(EV), INVSc1-TE(-), INVSc1-EE(+), and INVSc1-IE(+) groups, respectively, at day 0;

^a,b^ Means within the same row without common superscripts differ significantly (*P*<0.05), ns at *P*>0.05; *significant at the 5.00% level.

**Table S5. The percentages of top 10 relative abundances at species level at day 7.**

|  | 7 day | | | | |  |  |
| --- | --- | --- | --- | --- | --- | --- | --- |
|  | 7A | 7B | 7C | 7D | 7E |  |  |
|  |  |  |  |  |  |  |  |
|  | Means of mixed samples | | | | | SEM | *P*-values |
| *Lactobacillus_reuteri_I5007* | 3.833^b^ | 10.999^a^ | 10.079^a^ | 9.437^a^ | 10.676^a^ | 1.252 | *** |
| *Lactobacillus_amylovorus* | 2.353^b^ | 7.328^a^ | 7.058^a^ | 7.870^a^ | 7.497^a^ | 0.873 | *** |
| *human_gut_metagenome* | 5.533^b^ | 7.263^a^ | 7.366^a^ | 7.025^a^ | 7.190^a^ | 0.768 | * |
| *Phascolarctobacterium_succinatutens_YIT_12067* | 2.766 | 2.575 | 2.627 | 2.730 | 2.843 | 0.282 | ns |
| *Clostridium_sp* | 5.099^a^ | 2.718^b^ | 3.018^b^ | 2.589^b^ | 3.094^b^ | 0.432 | ** |
| *Lactobacillus_johnsonii* | 2.286^c^ | 3.668^ab^ | 4.131^a^ | 3.986^a^ | 3.531^b^ | 0.427 | * |
| *Ruminococcus_sp* | 2.145 | 2.467 | 2.230 | 2.512 | 2.151 | 0.318 | ns |
| *Lactobacillus_mucosae* | 1.335^b^ | 3.976^a^ | 4.071^a^ | 3.726^a^ | 3.832^a^ | 0.423 | ** |
| *Eubacterium_coprostanoligenes* | 0.913^b^ | 1.614^a^ | 1.689^a^ | 1.724^a^ | 1.500^a^ | 0.913 | *** |
| *wallaby_gut_metagenome* | 2.111 | 2.127 | 2.066 | 2.271 | 2.343 | 0.318 | ns |

**^1^Note:** 7A, 7B, 7C, 7D, and 7E represent the samples of the control, INVSc1(EV), INVSc1-TE(-), INVSc1-EE(+), and INVSc1-IE(+) groups, respectively, at day 7;

^a,b,c^ Means within the same row without common superscripts differ significantly (*P*<0.05), ns at *P*>0.05; *significant at the 5.00% level;**significant at the 1.00% level;***significant at the 0.10% level.

**Table S6. The percentages of top 10 relative abundances at species level at day 14.**

|  | 14 day | | | | |  |  |
| --- | --- | --- | --- | --- | --- | --- | --- |
|  | 14A | 14B | 14C | 14D | 14E |  |  |
|  |  |  |  |  |  |  |  |
|  | Means of mixed samples | | | | | SEM | *P*-values |
| *Lactobacillus_reuteri_I5007* | 1.295^b^ | 3.752^a^ | 3.492^a^ | 4.027^a^ | 3.828^a^ | 0.413 | ** |
| *Lactobacillus_amylovorus* | 0.845^b^ | 3.712^a^ | 3.557^a^ | 3.839^a^ | 3.377^a^ | 0.254 | *** |
| *human_gut_metagenome* | 1.818^c^ | 4.824^a^ | 4.133^b^ | 4.518^ab^ | 4.990^a^ | 0.329 | *** |
| *Phascolarctobacterium_succinatutens_YIT_12067* | 5.830 | 6.095 | 6.049 | 6.297 | 5.756 | 0.522 | ns |
| *Clostridium_sp* | 4.250^a^ | 1.141^b^ | 1.326^b^ | 1.272^b^ | 1.096^b^ | 0.136 | *** |
| *Lactobacillus_johnsonii* | 3.735^b^ | 5.593^a^ | 5.294^a^ | 5.242^a^ | 4.923^a^ | 0.472 | * |
| *Ruminococcus_sp* | 3.645 | 3.311 | 3.404 | 2.987 | 3.028 | 0.273 | ns |
| *Lactobacillus_mucosae* | 0.250^c^ | 0.903^a^ | 0.639^b^ | 0.671^b^ | 0.715^b^ | 0.072 | * |
| *Eubacterium_coprostanoligenes* | 1.048^b^ | 3.487^a^ | 3.730^a^ | 3.819^a^ | 3.857^a^ | 0.205 | * |
| *wallaby_gut_metagenome* | 0.682^b^ | 1.254^a^ | 1.338^a^ | 1.310^a^ | 1.240^a^ | 0.103 | * |

**^1^Note:**14A, 14B, 14C, 14D, and 14E represent the samples of the control, INVSc1(EV), INVSc1-TE(-), INVSc1-EE(+), and INVSc1-IE(+) groups, respectively, at day 14;

^a,b,c^ Means within the same row without common superscripts differ significantly (*P*<0.05), ns at *P*>0.05; *significant at the 5.00% level;**significant at the 1.00% level;***significant at the 0.10% level.

**Table S7. The percentages of top 10 relative abundances at species level at day 21.**

|  | 21 day | | | | |  |  |
| --- | --- | --- | --- | --- | --- | --- | --- |
|  | 21A | 21B | 21C | 21D | 21E |  |  |
|  |  |  |  |  |  |  |  |
|  | Means of mixed samples | | | | | SEM | *P*-values |
| *Lactobacillus_reuteri_I5007* | 0.419^b^ | 1.759^a^ | 1.605^a^ | 1.950^a^ | 2.093^a^ | 0.167 | ** |
| *Lactobacillus_amylovorus* | 0.598^c^ | 2.377^b^ | 2.464^b^ | 3.095^a^ | 2.532^b^ | 0.195 | *** |
| *human_gut_metagenome* | 1.203^b^ | 3.975^a^ | 3.675^a^ | 3.439^a^ | 4.085^a^ | 0.319 | ** |
| *Phascolarctobacterium_succinatutens_YIT_12067* | 1.047 | 0.865 | 0.942 | 1.132 | 1.099 | 0.111 | ns |
| *Clostridium_sp* | 4.121^a^ | 1.080^b^ | 1.177^b^ | 1.014^b^ | 1.108^b^ | 0.084 | *** |
| *Lactobacillus_johnsonii* | 3.987^b^ | 6.809^a^ | 7.305^a^ | 7.032^a^ | 6.908^a^ | 0.837 | ** |
| *Ruminococcus_sp* | 3.106 | 2.957 | 3.159 | 3.263 | 3.395 | 0.265 | ns |
| *Lactobacillus_mucosae* | 0.365^b^ | 1.080^a^ | 1.179^a^ | 1.139^a^ | 1.059^a^ | 0.083 | ** |
| *Eubacterium_coprostanoligenes* | 0.598 | 0.648 | 0.619 | 0.595 | 0.617 | 0.072 | ns |
| *wallaby_gut_metagenome* | 0.618^b^ | 1.185^a^ | 1.132^a^ | 1.063^a^ | 1.198^a^ | 0.081 | * |

**^1^Note:** 21A, 21B, 21C, 21D, and 21E represent the samples of the control, INVSc1(EV), INVSc1-TE(-), INVSc1-EE(+), and INVSc1-IE(+) groups, respectively, at day 21.

^a,b,c^ Means within the same row without common superscripts differ significantly (*P*<0.05), ns at *P*>0.05; *significant at the 5.00% level;**significant at the 1.00% level;***significant at the 0.10% level.

**
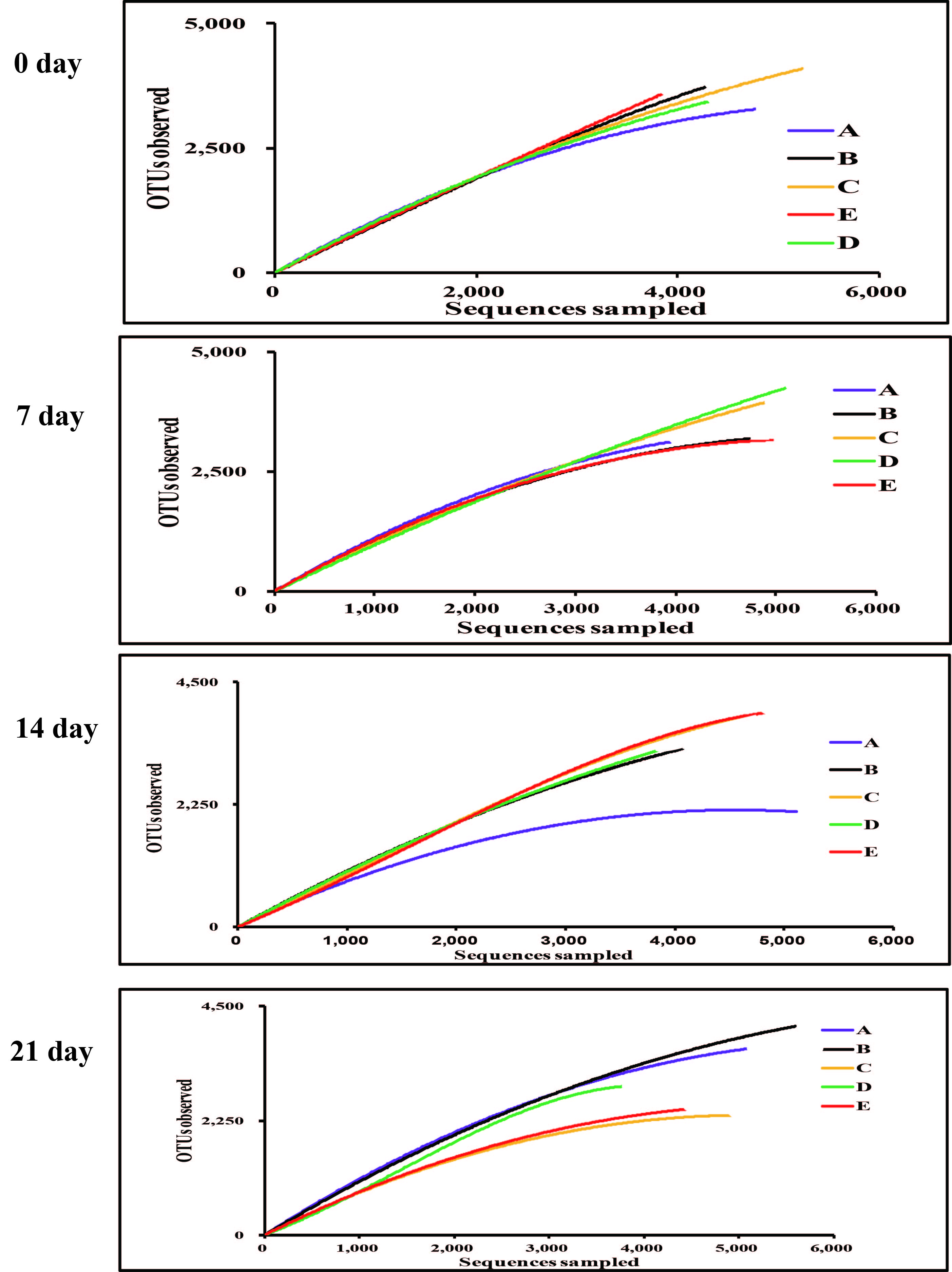
**

**Figure S1. Rarefaction curves for the animals at day 0, 7, 14 and 21.**

Note: A= Control group; B= INVSc1(EV) group; C= INVSc1-TE(-) group; D= INVSc1-EE(+) group; E=INVSc1-IE(+) group.
